# How does Information Exposure Affect Public Attitudes Toward GMO in China? The mediating and moderating roles of Conspiracy Belief and Knowledge

**DOI:** 10.3389/fpsyg.2022.955541

**Published:** 2022-09-20

**Authors:** Zhitao Du, Yuqi Xiao, Jinghong Xu

**Affiliations:** ^1^School of Journalism and Communication, University of Chinese Academy of Social Sciences (UCASS), Beijing, China; ^2^School of Journalism and Communication, Beijing Normal University, Beijing, China

**Keywords:** attitudes, GMO (genetically modified organism), information exposure, conspiracy theories belief, knowledge

## Abstract

**Background:**

In China, controversy about genetically modified organisms (GMO) is ongoing and some regard GMO as a “product of a conspiracy,” which affects people’s attitudes (PAs) toward GMO. Beliefs in conspiracy theories (BCT) are formed from the information that people are exposed to. Information exposure not only constructs a pseudo-environment for individuals to perceive the world, but also generates external stimuli for their mental states and attitudes. People’s objective knowledge and self-assessed knowledge play an important moderating role in this process.

**Method:**

The study adopted the stimulus-organism-response (SOR) model, with conspiracy beliefs as mediating variables, to test the mechanism of the independent variable of information exposure on the dependent variable of PAs toward GMO. Objective knowledge and self-assessed knowledge were introduced as moderator variables to explore the different roles of knowledge. A survey of Chinese adults was conducted in February 2022, and partial least squares structural equation modeling (PLS-SEM) was employed to estimate the multi-construct relationships.

**Results:**

Information exposure was significantly and directly connected with PAs toward GMO. BCT also played a significant mediating role. Unofficial information exposure reinforced beliefs in conspiracy theories. Stronger beliefs in conspiracy theories reduced people’s willingness to consume GMO foods and made them pessimistic about the development prospects of GMO foods. In contrast, exposure to official information weakened people’s beliefs in conspiracy theories and increased their willingness to consume GMO foods. In addition, the level of knowledge had a moderating role. Individual’s objective knowledge can effectively reduce the negative relationship of conspiracy beliefs on attitudes toward GMO development. Conversely, individual’s self-assessed knowledge can enhance the negative relationship of conspiracy beliefs on attitudes toward GMO development.

**Conclusion:**

Based on psychological and cognitive dimensions, this study provides a new perspective on how information exposure and people’s attitudes toward GMO are related to each other and enriches the variable measurement dimension of knowledge. Simultaneously, it provides a localized explanation of the factors affecting people’s attitudes toward GMO in China, providing a new theoretical basis for the subsequent development strategy of GMO foods.

## Introduction

As a technological innovation, genetically modified organism (GMO) is an important means of solving the global food shortage. China introduced the herbicide-resistant soyabeans in1996 and now GMO cultivation is a national policy (Chen, 2010). Although in the early 2000s, China was the third-largest global producer of GMO crops, following the United States and Argentina, GMO has become very controversial now. For example, the “golden rice” incident in late 2012 was so controversial that government officials avoided mentioning it at that time because it was too “sensitive” (Qiu, 2012). The dispute between Cui Yongyuan and Fang Zhouzi further sparked public backlash against GMO. Simultaneously, conspiracy theories have gradually spread among the Chinese people, including “Chinese children are being used in experiments on genetically modified rice” and “genetically modified food is being used as a biological weapon against China.” This series of incidents and rumors exacerbated the perceived problem of GMO in China and hindered its development. As of 2018, China ranked the seventh regarding the global GMO crop-growing countries. More importantly, the Chinese attitude toward GMO has changed significantly. Tao and Chen (2016) compared Chinese netizens’ attitude before and after the dispute between Cui and Fang to find that it changed from neutral to opposing GMO. In a sense, the advancement of genetic technology and GMO are dependent on public attitudes (PAs). Therefore, research on PAs toward GMO and the related factors should be examined further.

Western studies have investigated the factors influencing PAs toward GMO (House et al., 2001; Costa-Font and Gil, 2009), mainly focusing on advanced economies. Since consumers’ attitudes toward GMO vary across cultures and geographic characteristics worldwide (Aleksejeva, 2014), it is crucial to study the formation mechanism of attitudes toward GMO among citizens from different cultures and geographical environments. Based on a non-Western context sample, this study investigates the factors that are related to Chinese consumers’ attitudes toward GMO.

The stimulus-organism-response (SOR) model contends that external stimuli trigger the audience’s attitude and has long been used to study attitude changes. Among the stimuli, information often plays a key role in attitude formation. According to the pseudo-environment theory, people live in a virtual environment constructed by information media, and perceive the world with the help of information media. Considering China’s media system, cultural environment, and the media events related to GMO, this study aims to explore how varied information exposures affect individuals’ attitudes toward GMO.

Roukis (2006) found that technological progress can lead to uncertainty and create an atmosphere conducive to conspiracy thinking. This has been demonstrated in China where beliefs in conspiracy theories shape perceptions of GMO and adversely affect the future applications of biotechnology. Therefore, this study incorporate beliefs in conspiracy theories into the research framework. Meanwhile, since the previous research have explored the importance of objective knowledge and self-assessed knowledge on public attitude in the topics of science and technology risk (Rozenblit and Keil, 2002; House et al., 2004; Knight, 2005; Fernbach et al., 2019), this paper also tries to discuss the role of objective knowledge and self-assessed knowledge in the relationship between conspiracy theory belief and PAs toward GMO.

This study focuses on three questions:

What role does different information exposure play in the formation of PAs toward GMO?
Does conspiracy theory belief mediate the relationship between information exposure and GMO attitudes?
What role does knowledge play in this process?

This study integrates information exposure and conspiracy beliefs into the SOR model to explain the formation of GMO attitudes among the Chinese public, and to reflect the relationship between the media environment, conspiracy theories, and controversial scientific issues in the Chinese context. It reveals the different roles that objective knowledge and self-assessed knowledge play in this process, and provides insights into the dissemination of GMO knowledge in China. In addition, this study may help westerners to better understand the information environment and the psychological and cognitive factors that shape the Chinese public’s GMO attitudes.

## Literature review and hypotheses

Although GMO has become public knowledge since the 1990s, debates about GMO foods and related technology have never stopped. Previous research showed that Americans generally have a more positive attitude toward GMO than Europeans (Robinson, 1997; Bredahl, 2001; Lusk et al., 2004). Curtis et al. (2004) found that consumers in developing countries had more positive views toward GMO foods than consumers in developed countries. However, public attitudes toward GMO are rather complicated in China. Huang et al. (2006) found that as many as 62% of respondents in China had a positive attitude toward GMO, which is significantly higher than in other countries. Conversely, a survey by Wang and Xue (2005) in Beijing showed that most people trust traditional products over GMO products. Deng and Hu (2019) showed that 55% of Chinese consumers oppose GMO. These results make it difficult to tell Chinese attitudes toward GMO and further research is needed.

### The role of information exposure in the formation of GMO attitudes

Hovland (1959) developed the persuasion model based on the theory of information transition and social judgment. He regarded attitude change as a process during which outside information affects an individual’s attitude. Previous research confirmed that information is closely related to the perception and acceptance of GMO (Lusk et al., 2004; Onyango and Nayga, 2004) and we believe that information plays a crucial role in the public’s attitudes toward GMO. Previous research examined the influence of the nature of the words used in GMO reports on public GMO attitudes and showed that negative information can exacerbate negative public attitudes while positive information can stimulate positive attitudes (Nickerson, 1998; Lusk et al., 2004; Rousu et al., 2005; Huffman et al., 2007; Hu and Zhou, 2009). It should be emphasized that the public referred to in this study is the non-scientist group, because scientists and non-scientists may have completely different attitudes toward some issues.

Researchers have explored the influence of different information sources on attitudes toward GMO technology. Frewer et al. (1998) and Nisbet et al. (2002) contended that television, radio, and newspapers are the main channels of information for people to make biotechnological decisions. Previous studies have also showed the differences in people’s access to GMO information. Pang (2020) found that public attitudes toward GMO correlate with their dependence on information channels. Zhang et al. (2016) and Deng and Hu (2019) pointed out that individual trust in sources is also an important factor affecting attitudes toward GMO and that trust in government agencies and GMO experts positively correlates with the public acceptance of GMO. However, people may present completely different, or even opposing, perceptions and attitudes while using the same media and different types of information are more accurate predictors. Therefore, this study explores whether different information exposure affects public attitudes toward GMO from the perspective of official vs. unofficial information.

In China, media dominated by the governments and conveying mainstream ideology and values are usually defined as mainstream media and are also regarded as an official source of information. Specifically, mainstream media mainly refers to the newspapers, radio, and television stations of the central, provincial, and municipal party committees. He et al. (2015) found that people who obtained GMO information through public channels had a more positive attitude toward GMO while information from official sources has played a positive role in the application of transgenic technology in China. Pang (2020) also found that authoritative information dissemination channels play important roles in influencing the public’s knowledge and attitude toward GMO. Thus, we propose the following hypothesis:

*H1a*: Official information exposure has a significant, positive relationship with public attitudes toward GMO development.

Previous research demonstrated that it is more effective to know how individuals feel about purchasing or using an item than simply understanding consumers’ evaluation of the item itself (Azjen, 1980). Therefore, in addition to examining the public’s perception of the development of GMO technology, the public’s attitude toward the consumption of GMO foods is an important factor. Thus, we propose the following hypothesis:

*H1b*: Official information exposure has a significant positive relationship with public attitudes toward GMO foods consumption.

Globally, many countries have reported the continued growth in social media as news sources (Newman et al., 2017) and most consumers obtain information about GMO *via* the internet (Cui and Shoemaker, 2018; Deng and Hu, 2019). Previous research examined the role of social media in news consumption and its potential impact on individual decision-making and behavior (Fletcher and Nielsen, 2018; Huber et al., 2019). On one hand, some scholars are excited about its positive impact, arguing that equal access and equality in information production and dissemination contribute to the formation and maturation of deliberative democracy (Rishel, 2011). On the other hand, scholars have shown that due to the lack of gatekeepers, fact-checking, and imperfect legal systems, social media has gradually become a hotbed of conspiracy theories and rumors (Bastani and Bahrami, 2020; Hameleers et al., 2020; Vraga et al., 2020, 2022). Thus, the dangers of unofficial sources of information began to emerge. Deng and Hu (2019) found that consumers who obtained information on GMO through the internet or WeChat were less likely to accept GMO than those who obtained information from other channels. Thus, we propose the following hypotheses:

*H2a*: Unofficial information exposure has a significant negative relationship with public attitudes toward GMO development.

*H2b*: Unofficial information exposure has a significant negative relationship with public attitudes toward GMO food consumption.

### The complexity of attitude formation: Belief in conspiracy theories as mediating variable

Since factors influencing audience attitudes are diverse and complex, it is impossible to examine the linear relationship between specific variables. Among the models that explore changes in audience attitudes, the stimulus–response (S-R) and knowledge-attitude-practice (KAP) models are the most popular. Based on the S-R model, the SOR model was proposed, which posits that the audience’s attitude is triggered by external stimuli, directly or indirectly affecting the audience’s physiological and psychological states (Woodworth and Schlosberg, 1954). This study adopts the SOR model to explore the complex mechanism how information exposure affects public attitudes and behaviors toward GMO since the KAP model ignores the influence of external environment on attitudes.

Extensive research found that the perceived safety perception of GMO (Huang et al., 2014), the level of knowledge about GMO (Simis et al., 2016), the level of social trust (Jennings and Russell, 2019; Zhao et al., 2020), and the conspiracy theory beliefs (Yang, 2013) are correlated with the formation of public attitudes toward GMO and the polarization of public GMO attitudes. Considering that conspiracy theories about GMO are very popular in China, we focus on the relationship between conspiracy beliefs and GMO attitudes.

Conspiracy theory is construed to explain major social and political events by a small group of people out of self-interest and against public interest (Goertzel, 1994; Douglas and Sutton, 2008; Uscinski and Parent, 2014; Green and Douglas, 2018). With the development of the internet and the rise of social media, conspiracy theories have developed into “a mainstream paradigm through which many people try to understand the world” (Bantimaroudis et al., 2020). Previous research has found conspiracism to be a largely consistent predictor of specific anti-science beliefs across various domains (Landrum and Olshansky, 2019). There are also many conspiracy theories regarding GMO. For example, GMO crops were used by Americans to conquer the world, making developing countries more dependent on United States weapons for agricultural inputs (Ermakova, 2005; Robin, 2014).In China, a study showed that 13.8% of respondents believed that GMO technology was a form of bioterrorism against China (Cui and Shoemaker, 2018). Furthermore, some GMO conspiracy theories use academic research to increase their validity (Seralini et al., 2012). Once conspiracy beliefs are solidified, regulatory measures to debunk them may not have the expected effect (Stojanov et al., 2015; Wood, 2016). The promotion and development of GMO technology will also be affected. The golden rice case is a typical example (Shan and Jin, 2012).

Oleksy et al. (2020) distinguished two types of conspiracy theories—general conspiracy theories and government-related conspiracy theories. Scholars found that individuals who believe in one specific conspiracy theory often believe in other conspiracy theories, even logically conflicting ones (Wood et al., 2012). This empirical evidence has led scholars to define belief in conspiracy theories as a distinct psychological characteristic (Jia and Luo, 2021; Yang et al., 2021). This means that people tend to achieve their interpretive goals by attributing significant political or social events to the secrete plans of powerful groups or individuals (Goertzel, 1994; Uscinski and Parent, 2014; Green and Douglas, 2018). This study tends to focus on the role of belief in conspiracy theories in the relationship between information exposure and PAs toward GMO.

Extreme attitudes are associated with conspiracy theory beliefs when it comes to issues such as vaccination (Jolley and Douglas, 2014a) and climate change (Jolley and Douglas, 2014b).Conspiracy theory beliefs stabilize the self and inner group by blaming others for adverse outcomes, thereby polarizing attitudes (Douglas et al., 2017). Yang (2013) found that the strength of beliefs in conspiracy theories was a significant predictor of intentions to consume GMO foods. Thus, we propose the following hypotheses:

*H3a*: Individual beliefs in conspiracy theories are significantly negatively related to public attitudes toward GMO development.

*H3b*: Individual beliefs in conspiracy theories have significant negative relationship with public attitudes toward GMO food consumption.

Existing studies have demonstrated significant associations between different information exposure and beliefs in conspiracy theories (Hollander, 2018; Mancosu and Vegetti, 2021; Xiao et al., 2021). Stempel et al. (2007) contended that individuals who access official and mainstream media are more reluctant to believe the conspiracy theories. Thus, we propose the following hypothesis:

*H4a*: Official information exposure is significantly negatively associated with public belief in conspiracy theories.

Many studies proved that most “famous” conspiracy theories were originally generated from and spread on social media (Pennycook et al., 2020). Allington et al. (2021) argued that individuals who use social media as a source of news or information have stronger beliefs about COVID-19-related conspiracy theories. Moreover, Hu (2016) showed that rumors about food safety accounted for 45% of all internet rumors, seriously affecting public trust. Thus, we propose the following hypothesis:

*H4b: Unofficial information exposure has a significant positive relationship with public belief in conspiracy theories.*

### Objective knowledge and self-assessed knowledge as moderator variables

Knowledge has led to polarized attitudes toward scientific and policy issues (Van der Linden et al., 2017), and while relative strengths and weaknesses vary across issues (Drummond and Fischhoff, 2017), such polarization is evident in the case of GMO (Fernbach et al., 2019). The scientific community believes that scientific knowledge promotes public acceptance of new technologies (Simis et al., 2016). Many empirical studies have demonstrated that scientific knowledge is positively correlated with public support for science and learning scientific knowledge can compensate for information asymmetry in transgenic technology, thereby awakening individuals’ attitudes toward transgenic technology based on objective cognition (Priest, 2000; Allum et al., 2008).

However, empirical evidence also suggests that the correlation or explanatory power between scientific knowledge and the perception and acceptability of GMO technology is weak and unstable (Gaskell et al., 2000; Brossard and Nisbet, 2007; Connor and Siegrist, 2010; Druckman and Bolsen, 2011; Mielby et al., 2013). In China, Lv (2009) found that education was significantly correlated with the acceptance of biotechnology applications in food or agriculture. This indirectly relates to the correlation between the level of GMO knowledge and respondents’ acceptance of GMO. As China’s GMO controversy intensifies, the Chinese public’s attitude toward GMO also changes. Some research demonstrated that the role of knowledge levels in public support for GMO is unclear. Cui and Shoemaker (2018) found that more educated individuals are more skeptical of GMO, which contradicts previous studies. This contradiction may be explained by the fact that the knowledge examined in prior studies does not sufficiently reflect controversial scientific and technological issues.

Previous studies measured scientific knowledge only by asking about knowledge or by measuring knowledge questions directly related to GMO. However, scientific knowledge varies by “scientific issue” (Allum et al., 2008). Therefore, the scientific principles cannot be used to explain the audience’s level of GMO knowledge directly and indiscriminately. As Miller (1996) pointed out that the scientific literacy scale has three dimensions: first, scientific knowledge, referring to the mastery of vocabulary and scientific terms sufficient to read different scientific viewpoints in the media; second, scientific method, referring to the process of scientific inquiry or reasoning and possessing a certain understanding of logic; third, understanding the relationship between science and society, which means that individuals have a certain degree of cognition about the impact of science and technology on individuals and society. Thus, the psychosocial elements that shape the knowledge-attitude link of GMO must be considered.

The research perspective on risk communication differs from that of scientific communication, in that it regards knowledge as the basis of “risk perception.” When judging the impact of controversial technologies on themselves or on society, individuals often use knowledge as a reference to reduce cognitive risks and ultimately, affecting their attitudes (You and Jin, 2020). This is closer to the concept of social influence in the three dimensions of scientific literacy. You and Jin (2020) reconstructed the GMO knowledge scale from both perspectives of science communication and risk communication. The influence of GMO knowledge on attitude and behavior was investigated from three perspectives: scientific principles, GMO development, and social influence.

Considering that different knowledge levels are significantly correlated with beliefs in conspiracy theories (Van Prooijen, 2017), individuals with higher knowledge levels have lower beliefs in conspiracy theories. Thus, we propose the following hypotheses:

*H5a*: Objective knowledge reduces the negative relationship between belief in conspiracy theories and attitudes toward GMO development.

*H5b*: Objective knowledge reduces the negative relationship between belief in conspiracy theories and attitudes toward GMO foods consumption.

Additionally, knowledge is not a one-dimensional structure. On the highly controversial issue of GMO, Previous research focuses on what people do know. However, it is also important to consider what they think they know (House et al., 2004; Knight, 2005), or the self-assessed knowledge. That is, existing research focuses on the relationship between objective knowledge and attitudes, while ignoring the self-assessed knowledge. When affected by the illusion of knowledge, people often cannot judge how much they do know, and thus overestimate their understanding of things (Sloman and Fernbach, 2018). The illusion is far stronger for explanatory knowledge than for many other kinds of knowledge (Rozenblit and Keil, 2002), such as facts, procedures, or narratives. The Dunning–Kruger effect shows that people who are relatively incompetent have the strongest tendency to overestimate their own competence (Van Prooijen and Krouwel, 2020). Fernbach et al. (2019) demonstrated that people with less knowledge of GMO believe that they know more about GMO. They examined the relationships between extremity of opposition to GM foods, objective knowledge, and self-assessed knowledge about GM foods, and found extremists will display low objective knowledge but high self-assessed knowledge, and that the gap between the two will grow with extremity. Thus, we propose the following hypotheses:

*H6a*: Self-assessed knowledge can enhance the negative impact of belief in conspiracy theories on attitudes toward GMO development.

*H6b*: Self-assessed knowledge can enhance the negative impact of belief in conspiracy theories on attitudes toward GMO foods consumption.

**Figure 1:**
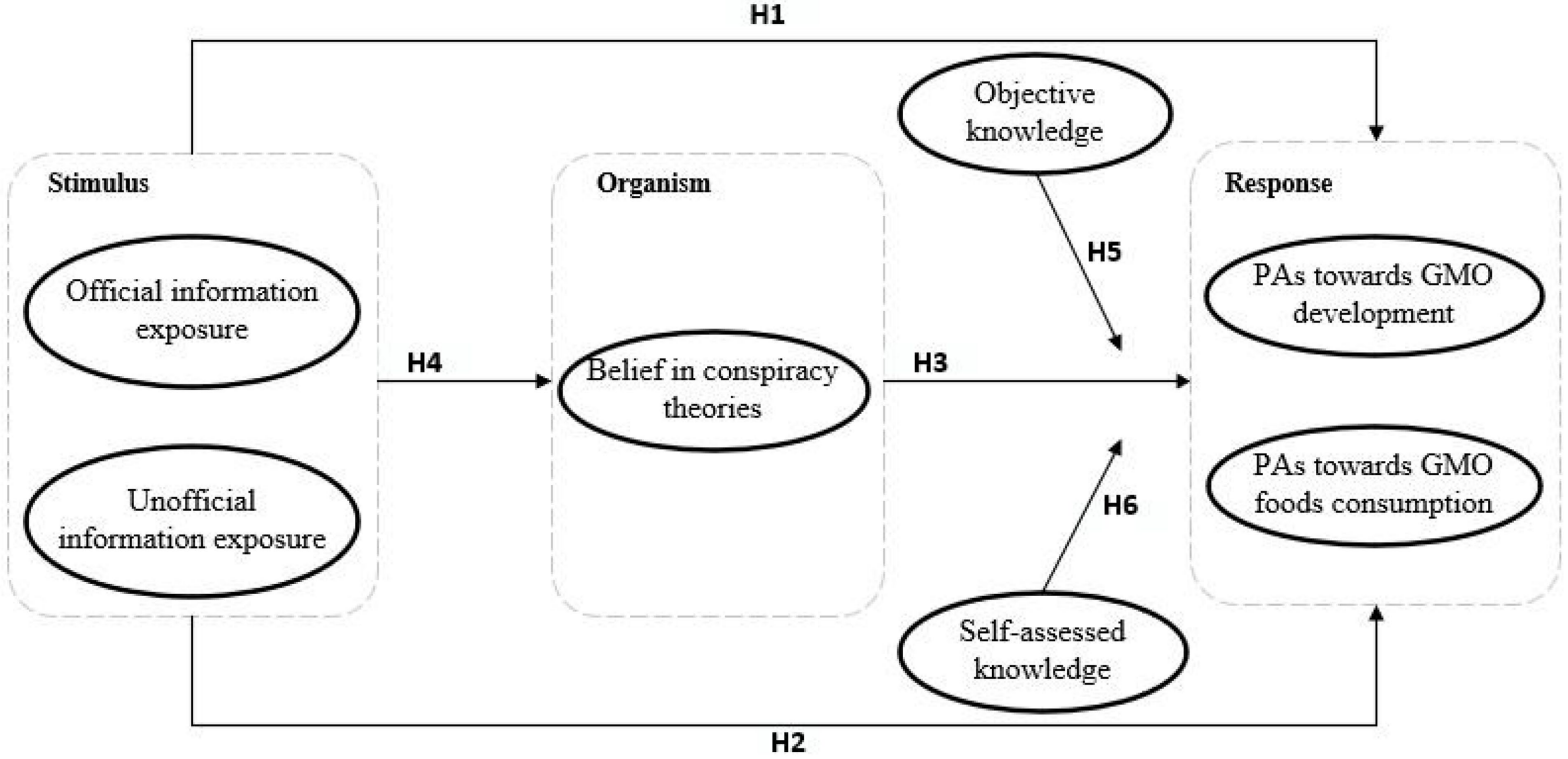
Research framework.

In summary, information exposure, beliefs in conspiracy theories, objective knowledge, and self-assessed knowledge are important variables that affect public attitudes toward GMO. These variables were integrated into the analytical framework and conceptual model of this study, as shown in Figure 1.

## Materials and methods

### Participants and procedures

This study conducted a cross-sectional online survey among Chinese adults from February 13, 2022, to March 25, 2022, using Sojump, a top Chinese professional online survey provider with a sampling service of 2.6 million registered users. In order to ensure the quality, we added screener questions and reverse questions. A total of 689 survey invitations to answer questionnaires were randomly sent out. We excluded unqualified questionnaires (those with less than 3 min to answer and those that did not pass the screener and reverse questions) and finally obtained a valid sample of 518, with an effective response rate of 75.2%. Upon completion, respondents received a gift of approximately one dollar as an incentive. Back-translation and pilot tests were conducted before the survey started. This study was approved by the Social Science Ethics Committee of a research university in Beijing, China (approval number: UCASS202201).

### Measurement

#### Information exposure

This study divides information exposure into official information exposure and unofficial information exposure by combining the research of Chu (2020) and Wang and Jin (2019). When investigating official information exposure, respondents were asked about the frequency of information they obtain on a daily basis from the following sources: (1) central media and their websites and APPs, including central-level media such as China Central Radio and Television, People’s Daily, Xinhua News Agency, and their accounts on social media; (2) local media and their websites and APPs, including provincial-, municipal-, and county-level radio and television stations, newspapers, and their accounts on social media. When investigating unofficial information exposure, respondents were asked through which of the following channels they primarily obtained unofficial information: (1) commercial and market-oriented news websites and their APPs; (2) WeChat; (3) Weibo; (4) relatives and friends; (5) online video/short video platforms; and (6) online forums communities. Each question was rated on a five-point Likert scale (1 = never, 2 = occasionally, 3 = sometimes, 4 = often, 5 = always).

#### Belief in conspiracy theories

The scale of belief in conspiracy theories (BCT) adopts the Universal Conspiracy Theory Mindset.

Scale by Imhoff and Bruder (2014). The questionnaire used a seven-point Likert scale (1 = completely disagree to 7 = strongly agree). There were 12 questions in this item, three of which had factor loadings below 0.7 and were removed from the model. The remaining 9 items included the following: “Those at the top can do whatever they want,” “A few powerful groups of people determine the destiny of millions,” “There are secret organizations that greatly influence political decisions,” “Politicians and other leaders are just string puppets of covert powers,” “Most people do not recognize the extent to which our life is determined by conspiracies that are concocted secretly,” “International intelligence agencies are involved in our everyday life to a much larger degree than people assume,” “Secret organizations can manipulate people psychologically so that they do not notice how their life is being controlled by others,” “There are certain political circles with secret agendas that are very influential,” “Most people do not see how much our lives are determined by plots hatched in secret,” and so forth. In the model, the seven-level scale was transformed into a five-level scale, and the conversion formula was 4 × (m − 1)/6 + 1, where m is the original value on the seven-level scale.

#### Knowledge

The knowledge dimension included two measurement variables: objective knowledge (OK) and self-assessed knowledge (SK). Self-assessed knowledge was measured by the question “How much do you think you know about genetically modified foods and related knowledge?” Responses were rated on a 5-point scale (1 = completely understand to 5 = completely unknown/I do not know). As mentioned above, the article draws on the research of You and Jin (2020) to measure the objective knowledge of GMO from three dimensions: “scientific principle,” “transgenic development status,” and “social impact.” First, “Scientific Principles” measured the audience’s understanding of the basic principles of GMO. The item consisted of four questions, including the understanding of hybrid breeding technology, genetic modification, agricultural biotechnology, and genetically modified food. Second, the “Current situation of genetic modification development” measured participants’ understanding of the current situation of genetically modified development. The topics included “China allows the cultivation of genetically modified food,” “China has mandatory regulations on the genetically modified food sold on the market, and must have a genetically modified label,” “The genetically modified crops approved for commercial planting in China include corn, soybeans, and potatoes,” and “China allows the seeds of genetically modified food crops to be imported for cultivation.” Third, “Social impact” assessed participants’ knowledge of the impact of genetic modification on society and the purpose of the development of genetically modified technology. The items included “GMO technology can improve crop yield,” “Eating genetically modified crops will seriously affect health,” “Growing genetically modified crops is harmful to the environment,” and “GMO technology can reduce the use of pesticides.” Each question of objective knowledge received 1 point, and the total score of objective knowledge was calculated. The following formula was used to convert the score into a 5-point system: 4 × (*n* − 1)/11 + 1, where n is the original score of objective knowledge.

#### Public attitudes toward GMO

The measurement of GMO attitude was divided into two variables: public attitude toward GMO development (PAGMD) and public attitude toward GMO food consumption (PAGMC). The questionnaire used a 5-point scale (1 = completely accepted, 5 = completely not accepted) to measure public attitudes toward GMO development based on the research of Xiang et al. (2005) and You and Jin (2020). The measurement questions included: “Do you support the research and development of GMO technology in China?” “Do you support the commercialization of GMO-related products in China?” and “Do you support the government’s use of GMO technology in the biomedical field?” Regarding assessing the variable of public attitude toward genetically modified food consumption, the questions were designed based on related questions in research questionnaires, such as INRA (2000) and Brossard and Nisbet (2007). Items included “Bread processed with genetically modified wheat resistant to diseases and insect pests.” “Rice produced from genetically modified rice resistant to pests and diseases.” “Rice produced from genetically modified rice providing improved nutrition.” “Will you accept eating genetically modified food?”

### Data analysis

Information on the demographic characteristics of the respondents was collected. The sample distribution was relatively balanced as shown in Table 1. The ratio of males to females was 44.6:55.4, which is roughly representative of the ratio of males to females in the overall population of China. Of the participants, 68.7% were between the ages of 25 and 35 years, and most had bachelor degrees, accounting for 78%. Occupationally, 78.4% of participants were enterprise managers and employees. At the same time, there were no university or research institution staff in the sample, which ensured that the study population was limited to non-scientists.

**Table 1:** The detailed demographic distribution.

	Characteristics	Frequency	Percent(%)
Gender	Male	231	44.6
	Female	287	55.4
Age	18–24	68	13.1
	25–30	216	41.7
	31–35	140	27.0
	36–40	45	8.7
	41–45	27	5.2
	46–50	14	2.7
	>51	8	1.6
Education level	Primary school or below	2	0.4
	Junior high school	2	0.4
	Senior high school	17	3.3
	Junior college	42	8.1
	Undergraduate degree	404	78.0
	Masters or higher	51	9.8
Occupation	Government	46	8.9
	Enterprise manager	117	22.6
	Employee	291	56.2
	Self-employed	40	7.7
	Peasant	7	1.4
	Other	17	3.3

Partial least squares structural equation modeling (PLS-SEM) was employed using SmartPLS 3.0 to estimate the simultaneous relationships among multiple constructs in this study. The model included both reflective constructs (PAs toward GMO development, PAs toward GMO food consumption, belief in conspiracy theories) and formative constructs (official information exposure and unofficial information exposure). PLS, a variance-based SEM, is preferred over the traditional covariance-based SEM for the current analysis.

Since the results may be susceptible to common method bias (CMB) when one respondent answers the questions in each questionnaire (Podsakoff et al., 2003), we created a common method factor (method construct) in the PLS model, including all indicators of the three principal constructs in the model (Liang et al., 2007). We then calculated the variances of each indicator, which were substantially explained by the corresponding principal and method constructs. The average substantive factor loading was 0.758, whereas the average method factor loading was 0.008, resulting in a ratio (substantive variance to method variance) of approximately 97:1. The loadings of the principal constructs were all significant (*p* < 0.01), while most loadings for the method were not significant (*p <* 0.05). In summary, the relatively small values of loadings and insignificance of the method variance suggest that CMB was not serious.

## Results

### Measurement model

The evaluation of the measurement model involved the assessment of reliability and validity for each reflective scale. Firstly, a reliability assessment was conducted. As shown in Table 2, Cronbach’s alpha and composition reliability (CR) values were both greater than 0.9, indicating good reliability.

**Table 2:** The convergent validity and reliability of reflective scales.

Constructs	Indicators	means	Standard deviations	Factor loading	Cronbach’s alpha	CR	AVE
BCT: belief in conspiracy theories	BCT2	3.256	0.914	0.805^***^	0.947	0.955	0.701
	BCT3	3.355	0.965	0.830^***^			
	BCT4	3.450	0.896	0.857^***^			
	BCT6	3.069	0.854	0.840^***^			
	BCT7	3.149	0.860	0.855^***^			
	BCT9	3.534	0.811	0.800^***^			
	BCT10	3.187	0.882	0.849^***^			
	BCT11	3.432	0.859	0.857^***^			
	BCT12	3.325	0.904	0.837^***^			
PAGMD: PAs toward GM development	PAGMD1	2.971	1.333	0.932^***^	0.928	0.954	0.875
	PAGMD2	3.083	1.410	0.940^***^			
	PAGMD3	3.089	1.424	0.934^***^			
PAGMC: PAs toward GM foods consumption	PAGMC1	2.959	1.039	0.805^***^	0.914	0.940	0.798
	PAGMC2	2.450	1.444	0.912^***^			
	PAGMC3	2.631	1.369	0.923^***^			
	PAGMC4	2.537	1.505	0.927^***^			

*** *p* < 0.001.

CR, composite reliability; AVE, average variance extracted.

Secondly, convergent validity was examined. Table 2 shows that the factor loadings based on confirmatory factor analysis (CFA) were above 0.7 and below 0.95, and the *p* values were all less than 0.05. Simultaneously, the average variance extraction (AVE) was greater than 0.7. Thus, the scale has convergent validity, according to the criteria proposed by Fornell and Larcker (1981).

Thirdly, we examined the discriminant validity. We first tested the cross-loading. Table 3 shows that the correlation coefficient between each measured variable and its latent variable (i.e., loading) was greater than the correlation coefficient between the measured variable and other latent variables (i.e., cross-loadings), indicating that the measurement model had good discriminant validity. We then compared the square root of the AVE and the correlation coefficient of each latent variable proposed by Fornell and Larcker (1981). As shown in Table 4, the value on the diagonal line is the square root of the AVE, and the value on the off-diagonal line represents the correlation coefficient of the latent variable. The former was larger than the latter. This description has good discriminant validity. We then used the HTMT.85 standard proposed by Henseler et al. (2015) to test the values in the obtained matrix to find that they were all less than 0.75, which indicates that each dimension had better discriminant validity.

**Table 3:** Loads and cross-loads of reflective variables.

	PAGMD	PAGMC	BCT
BCT2	−0.575	−0.474	0.805
BCT3	−0.573	−0.498	0.830
BCT4	−0.569	−0.474	0.857
BCT6	−0.633	−0.499	0.840
BCT7	−0.626	−0.504	0.855
BCT9	−0.512	−0.471	0.800
BCT10	−0.653	−0.531	0.849
BCT11	−0.587	−0.491	0.857
BCT12	−0.607	−0.502	0.837
PAGMD1	0.932	0.632	−0.666
PAGMD2	0.940	0.671	−0.674
PAGMD3	0.934	0.619	−0.651
PAGMC1	0.622	0.805	−0.477
PAGMC2	0.619	0.927	−0.550
PAGMC3	0.617	0.912	−0.522
PAGMC4	0.592	0.923	−0.557

**Table 4:** Correlation matrix of the reflective constructs.

	PAGMD	PAGMC	BCT
PAGMD	**0.935**		
PAGMC	0.686	**0.893**	
BCT	−0.710	−0.590	**0.837**

The numbers in bold on the matrix of correlation are the square roots of The AVE.

The test results of the formative variables are listed in Table 5. For official information exposure, only the weight of official information exposure in Central media and their websites and APPs and their accounts on social media was more than 0.2 and significant. The weight of official information exposure in local media and their websites and APPs and their accounts on social media was less than 0.2 and was not significant. This indicates that the central media is the main channel for Chinese people to obtain information. The weights of unofficial information exposure in WeChat, Weibo, and online video/short video platforms were more than 0.2 and were significant. In this study, indicators with weights greater than 0.2 and significant were included in the model. In addition, the VIF value of each index was less than 5, indicating that the collinearity problem was negligible.

**Table 5:** Assessment of formative constructs.

Constructs	Indicators	Means	Standard deviations	Weights
OI: official information	Central media and their websites and apps and their accounts on social media	2.595	1.569	0.836^***^
	Local media and their websites and apps and their accounts on social media	2.560	1.579	0.180
UI: unofficial Information	Commercial and market-oriented news websites and their apps	3.145	1.349	−0.275
	WeChat	3.259	1.964	0.582^***^
	Weibo	3.438	1.191	0.331^**^
	Relatives and friends	3.102	1.001	−0.127
	Online video/short video platforms	2.956	1.072	0.303^**^
	Online forums communities	3.263	1.221	0.042

** *p* < 0.001;

*** *p* < 0.001.

### Structural model

In PLS analysis, the path relationship formed by the constructs constitutes the structural model. PLS uses the bootstrap method to test the significance of the path relationships. Based on Chin and Newsted (1999), this study set the number of subsamples to 5,000 to achieve a stable estimation of the parameters.

#### Direct effect analysis

In Table 6, Model 1 shows that official information exposure had a significant positive relationship with.

**Table 6:** Path coefficients of the structural model.

	**Model 1**		**Model 2**		**Model3**		**Model 4**	
	**PAGMD**	**PAGMC**	**PAGMD**	**PAGMC**	**PAGMD**	**PAGMC**	**PAGMD**	**PAGMC**
	R^2^ = 0.306, Q^2^ = 0.264	R^2^ = 0.256, Q^2^ = 0.200	R^2^ = 0.527, Q^2^ = 0.457	R^2^ = 0.381, Q^2^ = 0.300	R^2^ = 0.752, Q^2^ = 0.651	R^2^ = 0.511, Q^2^ = 0.400	R^2^ = 0.647, Q^2^ = 0.552	R^2^ = 0.523, Q^2^ = 0.412
OI	0.354^***^(0.039)	0.346^***^(0.042)	0.105^**^(0.035)	0.155^***^(0.043)	0.139^***^(0.024)	0.185^***^(0.040)	0.105^**^(0.031)	0.145^***^(0.041)
UI	−0.310^**^(0.040)	−0.258^***^(0.043)	−0.121^**^(0.038)	−0.112^**^(0.040)	−0.099^***^(0.026)	−0.094^*^(0.038)	−0.105^**^(0.032)	−0.081^*^(0.036)
BCT			−0.596^***^(0.032)	−0.454^***^(0.038)	−0.284^***^(0.034)	−0.219^***^(0.052)	−0.492^***^(0.037)	−0.243^***^(0.041)
OK					0.538^***^(0.031)	0.417^***^(0.051)		
OK × BCT					0.097^**^(0.032)	0.027 (0.045)		
SK							−0.308^***^(0.032)	−0.438^***^(0.038)
SK × BCT							−0.214^***^(0.032)	−0.036 (0.030)
			**BCT**		**BCT**		**BCT**	
			R^2^ = 0.368, Q^2^ = 0.255		R^2^ = 0.511, Q^2^ = 0.353		R^2^ = 0.486, Q^2^ = 0.334	
OI			−0.413^***^ (0.037)		−0.332^***^(0.036)		−0.321^***^(0.035)	
UI			0.308^***^ (0.039)		0.220^***^(0.041)		0.224^***^(0.036)	
OK					−0.408^***^(0.041)			
SK							0.356^***^(0.031)	

* *p* < 0.05;

** *p* < 0.01;

*** *p* < 0.001.

The numbers in brackets are standard deviations. EF(f^2^) is in the acceptable range. The data in parentheses after the path coefficients are bootstrap standard errors. OI, official information exposure; UI, unofficial information exposure; BCT, belief in conspiracy theories; PAGMD, PAs toward GMO development; PAGMC, PAs toward GMO food consumption.

PAs toward GMO development (*β* = 0.354, *p <* 0.001). Moreover, there was a significant positive relationship between official information exposure and PAs toward GMO food consumption (*β* = 0.346, *p <* 0.001), validating hypotheses H1 (a) and H1 (b). Unofficial information exposure had a significant negative relationship with PAs toward GMO development (*β* = *−*0.310*, p <* 0.01). Additionally, there was a significant negative relationship between unofficial information exposure and PAs toward GMO food development (*β* = *−*0.258, *p <* 0.001), validating hypotheses H2 (a) and H2 (b). Official information exposure had a positive relationship with people’s attitude toward the development and consumption of GMO foods. Furthermore, unofficial information exposure had a negative relationship with people’s attitude toward the development and consumption of GMO foods.

#### Mediating effect analysis

To explore the impact mechanism of information exposure on PAs toward GMO, it is necessary to analyze the indirect effects by mediating variables. Therefore, this study added belief in conspiracy theories as an intermediary variable in Model 1, as shown in Model 2 in Table 6. Belief in conspiracy theories on public GMO development attitude (*β* = *−*0.596*, p <* 0.001) and public GMO food consumption attitudes (*β* = *−*0.454*, p <* 0.001) had a significant negative relationship, which verified hypotheses H3 (a) and H3 (b). In addition, there was a significant negative relationship between official information exposure and beliefs in conspiracy theories (*β* = *−*0.413*, p <* 0.001). Moreover, unofficial information exposure and beliefs in conspiracy theories showed a significant positive relationship (*β* = 0.308*, p <* 0.001).

This study used the bootstrap method to test the mediation effects. As shown in Table 7, beliefs in conspiracy theories played a significant mediating role in the relationship between official information exposure and GMO food consumption attitudes. The indirect effect on the relationship between official information exposure and GMO development attitudes was 0.246, accounting for 70% of the total effect. The indirect effect on the relationship between official information exposure and GMO food consumption attitudes was 0.188, accounting for 54.8% of the total effect.

**Table 7:** Significance analysis of the mediation effects.

Path	Indirect effect	Direct effect	Total effect	Indirect effect/total effect
OI → BCT → PAGMD	0.246^***^ t = 10.222; *p* = 0.000; [0.194,0.287]	0.105^**^ t = 2.974; *p* = 0.003; [0.042,0.178]	0.351^***^ t = 8.904; *p* = 0.000; [0.272,0.428]	70.0%
OI → BCT → PAGMC	0.188^***^ t = 8.055; *p* = 0.000; [0.141,0.231]	0.155^***^ t = 3.633; *p* = 0.000; [0.073,0.263]	0.343^***^ t = 7.380; *p* = 0.000; [0.244,0.426]	54.8%
UI → BCT → PAGMD	−0.183^***^ t = 7.172; *p* = 0.000; [−0.239,-0.141]	−0.121^**^ t = 3.155; *p* = 0.002; [−0.200,-0.052]	−0.304^***^ t = 7.194; *p* = 0.000; [−0.387,-0.229]	60.2%
UI → BCT → PAGMC	−0.140^***^ t = 6.407; *p* = 0.000; [−0.184,-0.102]	−0.112^**^ t = 2.798; *p* = 0.005; [−0.199,-0.043]	−0.252^***^ t = 5.612; *p* = 0.000; [−0.355,-0.175]	55.6%

** *p* < 0.001;

*** *p* < 0.001.

OI, official information exposure; UI, unofficial information exposure; BCT, belief in conspiracy theories; PAGMD, PAs toward GMO development; PAGMC, PAs toward GMO food consumption.

Beliefs in conspiracy theories also played a significant mediating role between unofficial information exposure and attitudes toward GMO foods consumption. The indirect effect on the relationship between unofficial information exposure and attitudes toward GMO development was −0.183, accounting for 60.2% of the total effect. The indirect effect on the relationship between unofficial information exposure and GMO food consumption attitude was −0.140, accounting for 55.6% of the total effect.

#### Moderating effect analysis

Based on the mediation model (Model 2), objective knowledge and self-assessed knowledge were added as moderating variables. First, when the moderating variable of objective knowledge was added to Model 2 as shown in Model 3 in Table 6, there was a significant positive relationship between objective knowledge and PAs toward GMO development (*β* = 0.538*, p <* 0.001). There was also a significant positive relationship between PAs toward GMO foods consumption (*β* = 0.417*, p <* 0.001). The interaction term between objective knowledge and belief in conspiracy theories had a significant positive effect on PAs toward GMO development (*β* = 0.097*, p <* 0.01). This indicates that higher objective knowledge can effectively reduce beliefs in the negative relationship of conspiracy theories on PAs toward GMO development. This confirmed hypothesis H5 (a). However, the same interaction term had no significant relationship on PAs toward GMO food consumption, and Hypothesis H5 (b) was not confirmed.

Second, when the moderating variable of self-assessed knowledge was added to Model 2 as shown in Model 4 in Table 6, self-assessed knowledge had a significant negative relationship with PAs toward GMO development (*β* = *−*0.308*, p <* 0.001). Simultaneously, there was also a significant negative relationship with PAs toward GMO foods consumption (*β* = *−*0.438*, p <* 0.001). The interaction term between self-assessed knowledge and belief in conspiracy theories had a significant negative relationship on PAs toward GMO food development (*β* = *−*0.214*, p <* 0.01). This indicates that higher self-assessed knowledge enhanced beliefs in conspiracy theories on the negative relationship of PAs toward GMO food development. This confirmed hypothesis H6 (a). However, the same interaction term had no significant relationship on PAs toward GMO food consumption, and Hypothesis H6 (b) was not confirmed.

## Conclusion and discussion

Based on the SOR model, this study explored the structural relationship between information exposure, beliefs in conspiracy theories, and Chinese consumers’ attitudes toward GMO. In addition, objective knowledge and self-assessed knowledge were introduced as moderator variables to explore the different influences formed by knowledge differences.

First, different types of information exposure have significantly different relationships with public attitudes toward GMO. The SOR model emphasizes that external stimuli trigger audience attitudes, and information as an important stimulus has been widely discussed in previous studies on GMO attitudes. However, previous studies have rarely compared the effects of different information exposure on GMO attitudes, and their research objects either focused on traditional media channels (Frewer et al., 1998; Pang, 2020) or social media (Zhu et al., 2018; Deng and Hu, 2019). Thus, there was a lack of integrated discussion of different information exposure. This study focused on exploring the differences in the influence of different types of information exposure on the formation of people’s attitudes. Moreover, we divided information exposure into official and unofficial information exposure and examined the role of different types of information exposure in the formation of attitudes toward GMO. Our results showed that official information exposure had a significant positive relationship and played a leading role in the development and consumption attitudes of the public toward GMO foods. This shows that in China, more individuals who obtain GMO information from official channels tend to have a more positive attitude toward GMO foods. This is consistent with our research hypothesis and confirms prior research which concluded that traditional media is the source of people’s acquisition of GMO information. Sources with high authority and reliability, and public channels, play an active and important role in influencing public attitudes toward GMO (Nisbet et al., 2002; He et al., 2015; Pang, 2020).

In China, central and local media such as radio, television, newspapers and their websites, APPs, and accounts on social media are spokespersons of government discourse and are responsible for publicizing policies, guiding ideology, establishing the national image, and maintaining social stability (Gan, 1994). Although there are disputes about GMO, promoting GMO development is a basic policy for the long-term development planned by the Chinese government. Since 2021, the 20th Meeting of the Central Committee for Comprehensively Deepening Reform and the Ministry of Agriculture and Rural Affairs of China have put forward guiding opinions and evaluations of the GMO issue. The official media will naturally guide the public to recognize and support GMO foods and the public will have a positive attitude toward the development and consumption of GMO foods.

In contrast, unofficial information in WeChat, Weibo, online videos, and short videos were negatively correlated with the development and consumption attitudes of the public toward GMO foods. This is consistent with the finding of Deng and Hu (2019) that consumers who obtained information through social media were less likely to accept GMO foods than those who obtained information from other channels.

Second, belief in conspiracy theories played an important mediating role in the relationship between information exposure and the GMO attitudes. This study incorporated into the SOR model, the belief in conspiracy theories regarding GMO, an important variable that is often mentioned but rarely studied, as a mediating variable in the research framework. This study found that beliefs in conspiracy theories were significantly negatively correlated with public attitudes toward GMO. This validated our hypothesis that the stronger the individual beliefs in conspiracy theories, the more negative their attitudes toward GMO. This corroborates the finding of Shan and Jin (2012) that beliefs in conspiracy theories have hindered the promotion of golden rice and provides additional empirical evidence. As an important predictor of public attitudes toward GMO, beliefs in conspiracy theories must be considered in subsequent studies.

Thus, individuals who were exposed to more official information decreased their beliefs in conspiracy theories and, consequently, had more positive attitudes toward GMO foods. Conversely, individuals who were exposed to more unofficial information strengthened their beliefs in conspiracy theories and, subsequently, had more negative attitudes toward GMO foods. This is consistent with our research assumptions and with previous research findings on the relationship between media exposure and beliefs in conspiracy theories (Hollander, 2018; Mancosu and Vegetti, 2021; Xiao et al., 2021).

Third, citizens’ objective knowledge and their self-assessed knowledge had different relationships with attitudes toward GMO. This study found that objective knowledge restrained the negative attitudes of beliefs in conspiracy theories toward GMO development. However, this inhibitory effect was not significant regarding the negative attitudes of beliefs in conspiracy theories toward GMO food consumption. This conclusion partially confirms our hypothesis and echoes the results of previous studies (Hudson et al., 2015; Vecchione et al., 2015; Van Prooijen, 2017). Compared with the attitude toward GMO technology, when people consume GMO foods, they will consider their own economic situation and many other factors. Siegrist and Cvetkovich (2000) argued that with limited personal knowledge, non-professionals mainly rely on social trust when judging the risks and benefits of new technology. This finding suggests that trust reduces the cost and complexity of making rational judgments based on knowledge. Many studies have proven that trust in the government, research institutions, scientific research institutions, and even media institutions have played an important role in accelerating people’s acceptance of GMO food (Lobb et al., 2007; Zhang et al., 2016) and this will be our direction of research in the future.

Zhu et al. (2018) indicated that, in China, objective knowledge rather than self-assessed knowledge plays a decisive role in the process of forming attitudes toward GMO foods. This study found that the role of self-assessed knowledge in the influence of beliefs in conspiracy theories on GMO food consumption attitudes is not significant. However, it plays a reinforcing role in the negative impact of beliefs in conspiracy theories on GMO development attitudes. This is especially true among individuals with higher self-assessed knowledge, whose beliefs in conspiracy theories have a greater negative impact on attitudes toward GMO development. This supports our research hypothesis and confirms previous findings that individuals’ extreme attitudes toward GMO development are not only related to their lower objective knowledge, but also to their higher self-assessed knowledge. Specifically, people who think that they have more knowledge are more likely to believe in conspiracy theories and are more negative toward the GMO development.

Instead of reducing the cognitive differences among people with different attitudes toward GMO crops, educating the public about GMO crops will lead to greater differences in attitudes between those who are extremely opposed to GMO crops and those who support them. Therefore, it is necessary to promote IH (Intellectual Humility) literacy (Davis et al., 2016), based on scientific communication to the general public, so as to enhance individual self-awareness, including openness, curiosity, and inclusiveness, and to reduce the emergence of extreme views (Leary et al., 2017).

This study had several limitations. First, considering the difficulty of data collection, the survey data had a cross-sectional design, which was insufficient to determine causality. Future studies should adopt a longitudinal design. Second, the context of this study is in China. Thus, researchers should be cautious when applying the conclusions to other contexts. In addition, variables such as income, education, social trust and Nationalism are important factors affecting individuals’ attitudes toward GMO, but we could not exhaust them all in one study.

## Data availability statement

The raw data supporting the conclusions of this article will be made available by the authors, without undue reservation.

## Ethics statement

This study was approved by the Social Science Ethics Committee of a research university in Beijing, China (approval number: UCASS202201).

## Author contributions

ZD: manuscript writing, data analysis, and manuscript revision. YX: literature review and manuscript writing and manuscript revision. JX: conceptualization and survey execution. All authors contributed to the article and approved the submitted version.

## Funding

This study was funded by the National Social Science Fund of China with the grant number of 20VYJ014 and Foundation of Chinese Academy of Social Science for Marxist Theory Discipline Construction and Theoretical Research with the grant number of 2018mgczd002.

## Conflict of interest

The authors declare that the research was conducted in the absence of any commercial or financial relationships that could be construed as a potential conflict of interest.

## Publisher’s note

All claims expressed in this article are solely those of the authors and do not necessarily represent those of their affiliated organizations, or those of the publisher, the editors and the reviewers. Any product that may be evaluated in this article, or claim that may be made by its manufacturer, is not guaranteed or endorsed by the publisher.
